# Isochronal late activation mapping of epicardial ventricular tachycardia in a patient with midventricular obstructive hypertrophic cardiomyopathy

**DOI:** 10.1016/j.hrcr.2022.03.001

**Published:** 2022-03-10

**Authors:** Yukimi Uotani, Yousaku Okubo, Yuki Komatsu, Akihiko Nogami, Kazutaka Aonuma, Yukiko Nakano

**Affiliations:** ∗ Department of Cardiovascular Medicine, Hiroshima University Graduate School of Biomedical and Health Sciences, Hiroshima, Japan; † Department of Cardiology, Faculty of Medicine, University of Tsukuba, Tsukuba, Japan

**Keywords:** Isochronal late activation mapping, Apical aneurysm, Ventricular tachycardia, MVOHCM, Entrainment pacing, Epicardial reentry tachycardia

## Introduction

Midventricular obstructive hypertrophic cardiomyopathy (MVOHCM) with left ventricular (LV) aneurysm is associated with a high prevalence of ventricular arrhythmias.^1^^,^^2^ A recent study on 15 patients with hypertrophic cardiomyopathy (HCM) and apical aneurysm (AA) who underwent radiofrequency catheter ablation (RFCA) of ventricular tachycardia (VT) demonstrated that a combination of endocardial and epicardial approaches were effective in suppressing VT, and successful RFCA site or critical isthmus was at the scar border zone of the aneurysmal neck.^3^ Since activation mapping is difficult to perform in hemodynamically unstable VT, substrate modification during sinus rhythm for critical isthmus sites has become the main ablation strategy for scar-related VTs.^4^

Recently, isochronal late activation mapping (ILAM) during sinus rhythm has been reported as a breakthrough technique to identify VT termination sites.^5^

This report presents a rare case of sustained VT in a patient with MVOHCM that was successfully treated with catheter ablation using the novel mapping technique ILAM.

## Case report

**Figure 1:**
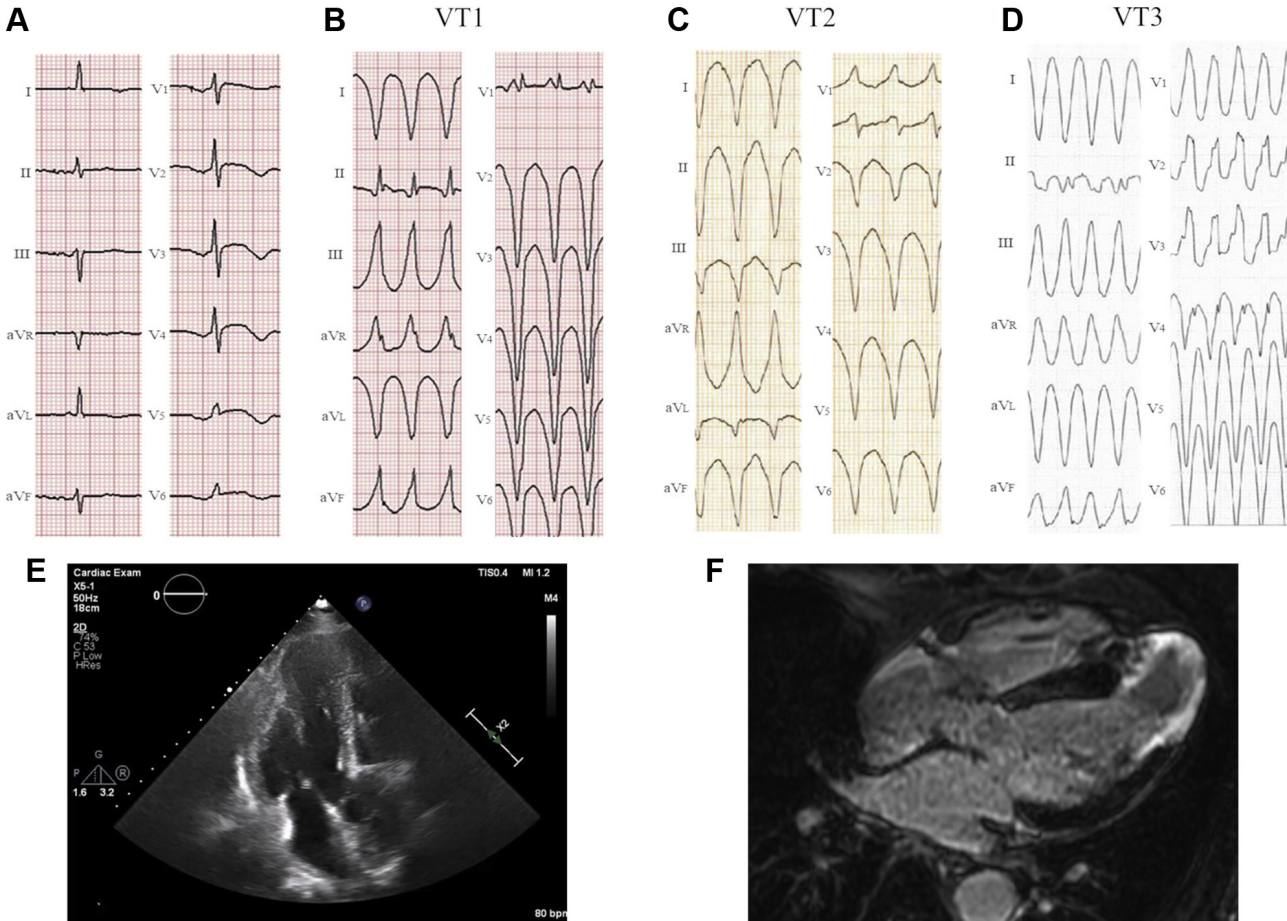
**A–D:** Twelve-lead electrocardiography (ECG) showing ventricular tachycardia (VT). **A:** ECG during normal sinus rhythm. **B:** ECG upon hospital admission showed sustained VT (VT1), which was a fast heart rate (200 beats/min) with right bundle branch block morphology and inferior axis. **C:** ECG at the previous admission showed a monomorphic VT (VT2) with right bundle branch block morphology and superior axis. **D:** ECG at the previous admission showed monomorphic VT (VT3) with right bundle branch block morphology and inferior axis. **E:** Cardiac echocardiography showed midventricular obstructive hypertrophy and large apical aneurysm (AA). **F:** Contrast and magnetic resonance imaging showed transmural delayed enhancement in the entire circumference of the AA.

A 70-year-old man with MVOHCM and AA was admitted to our hospital for VT recurrence. He had multiple VTs and a medical history of treatment with antiarrhythmic drugs (amiodarone 200 mg/day and bisoprolol 2.5 mg/day) and implantable cardioverter-defibrillator implantation. Twelve-lead electrocardiography upon admission showed a sustained VT (VT1), which was a fast heart rate (200 beats/min) with right bundle branch block morphology and inferior axis (Figure 1B). The QRS morphologies of the sustained VT1 were different from those of other VTs (VT2 and VT3) observed in previous hospitalizations (VT2: Figure 1C, VT3: Figure 1D). Figure 1A shows the electrocardiogram during normal sinus rhythm. Cardiac echocardiography showed midventricular obstructive hypertrophy and large AA (Figure 1E). Contrast magnetic resonance imaging revealed a transmural delayed enhancement in the entire AA circumference (Figure 1F).

**Figure 2:**
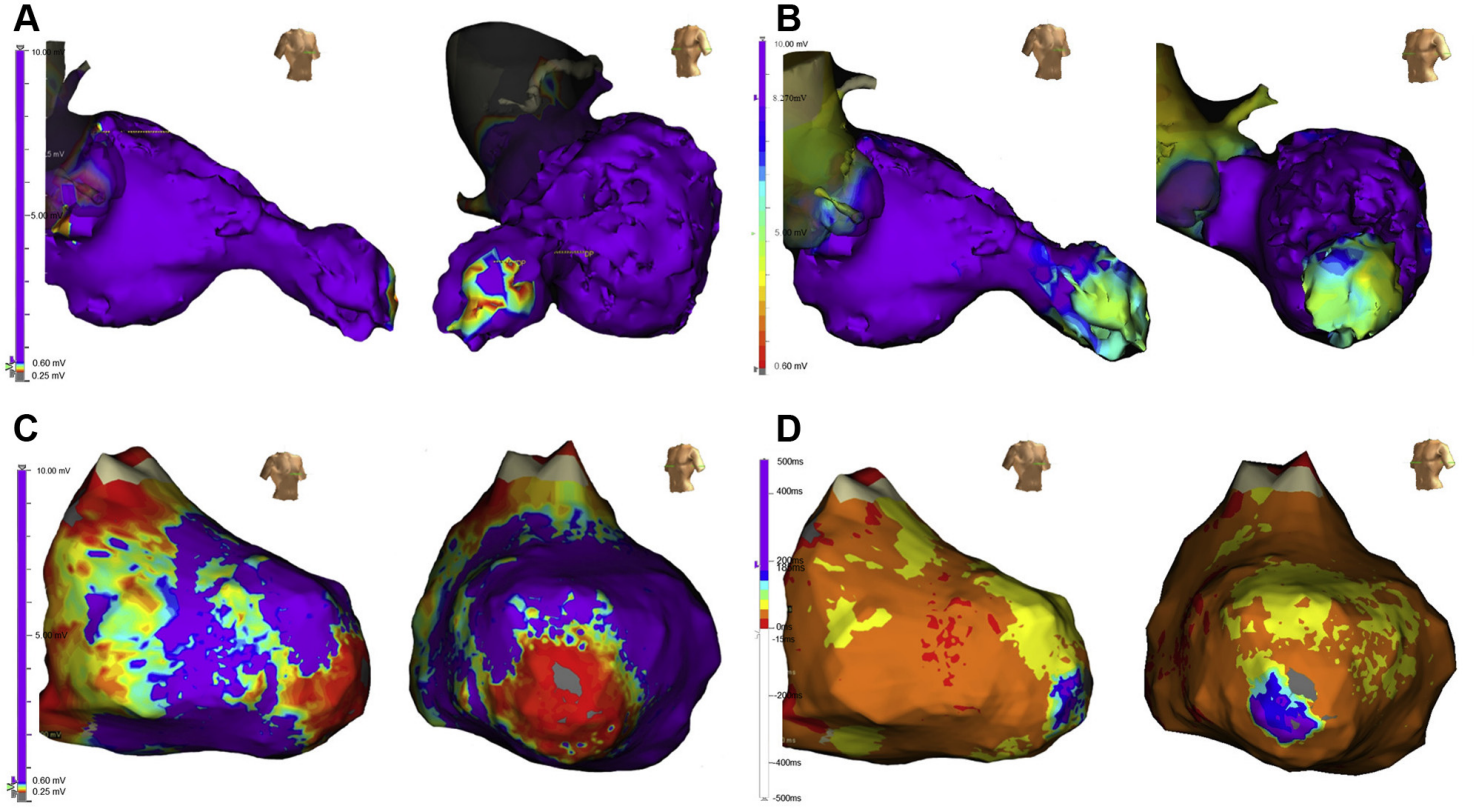
**A:** Endocardial left ventricular (LV) bipolar voltage mapping during sinus rhythm. The low-voltage and dense scar areas were defined as areas with peak-to-peak bipolar voltages of 0.1–0.6 mV and <0.1 mV, respectively. The voltage map showed low-voltage area at the anterior wall of the apical aneurysm (AA) neck side only. **B:** Endocardial LV unipolar voltage mapping during sinus rhythm. The normal signal amplitude was defined as ≥8.27 mV. The voltage map revealed the extensive voltage abnormality in the entirety of the LV aneurysmal area. The abnormal signal area in the endocardial voltage map was well correlated with that in the epicardial bipolar voltage map. **C:** Epicardial LV voltage mapping during sinus rhythm. The low-voltage and dense scar areas were defined as areas with peak-to-peak bipolar voltages of 0.1–0.6 mV and <0.1 mV, respectively. The voltage map showed a wide area with low voltages around the AA, where late potentials were observed. **D:** Epicardial LV isochronal late activation map (ILAM) during sinus rhythm. ILAM was displayed in 8 equally distributed isochrones of activation. ILAM showed the deceleration zone (DZ) around the LV apex, defined as a slow conduction region, and conduction velocity in the DZ is estimated as <0.6 m/s.

First, endocardial catheter ablation was performed. Endocardial LV voltage mapping during sinus rhythm was performed using a duodecapolar electrode catheter (Inquiry^TM^ Livewire^TM^; Abbott, St. Paul, MN) and EnSite^TM^ NavX^TM^ systems (Abbott, St. Paul, MN). The low-voltage and dense scar areas were defined as areas with peak-to-peak bipolar voltages of 0.1–0.6 and <0.1 mV, respectively. The bipolar voltage map showed the low-voltage area only on the anterior wall of the AA neck side but did not show a clear late potential (Figure 2A). However, endocardial LV unipolar voltage mapping showed extensive voltage abnormality in the entirety of the LV aneurysmal area (Figure 2B). Since the clinical VT (VT1) induced by burst right ventricular (RV) pacing was hemodynamically unstable, we could not create a VT activation map. Therefore, an 8F, 4-mm flexible irrigated-tip catheter (FlexAbility Ablation Catheter; Abbott, St. Paul, MN) was placed on the site, showing better pace mapping on a low-voltage area at the anterior and anteroseptal scar border in the AA. The application of radiofrequency energy to this site terminated the VT and suppressed it for just a short while, but clinical VT (VT1) immediately recurred by the RV burst pacing. Since the QRS morphology of VT1 was suggestive of epicardial origin, the epicardial approach was attempted. An epicardial ventricular voltage map was created using a high-density mapping catheter (Advisor^TM^ HD Grid Mapping Catheter; Abbott, St. Paul, MN) and EnSite NavX systems (Abbott, St. Paul, MN). The voltage map showed a wide area with low voltages around the AA, where late potentials were observed (Figure 2C). Interestingly, the abnormal signal area in the endocardial unipolar voltage map was well correlated with that in the epicardial bipolar voltage map.

**Figure 3:**
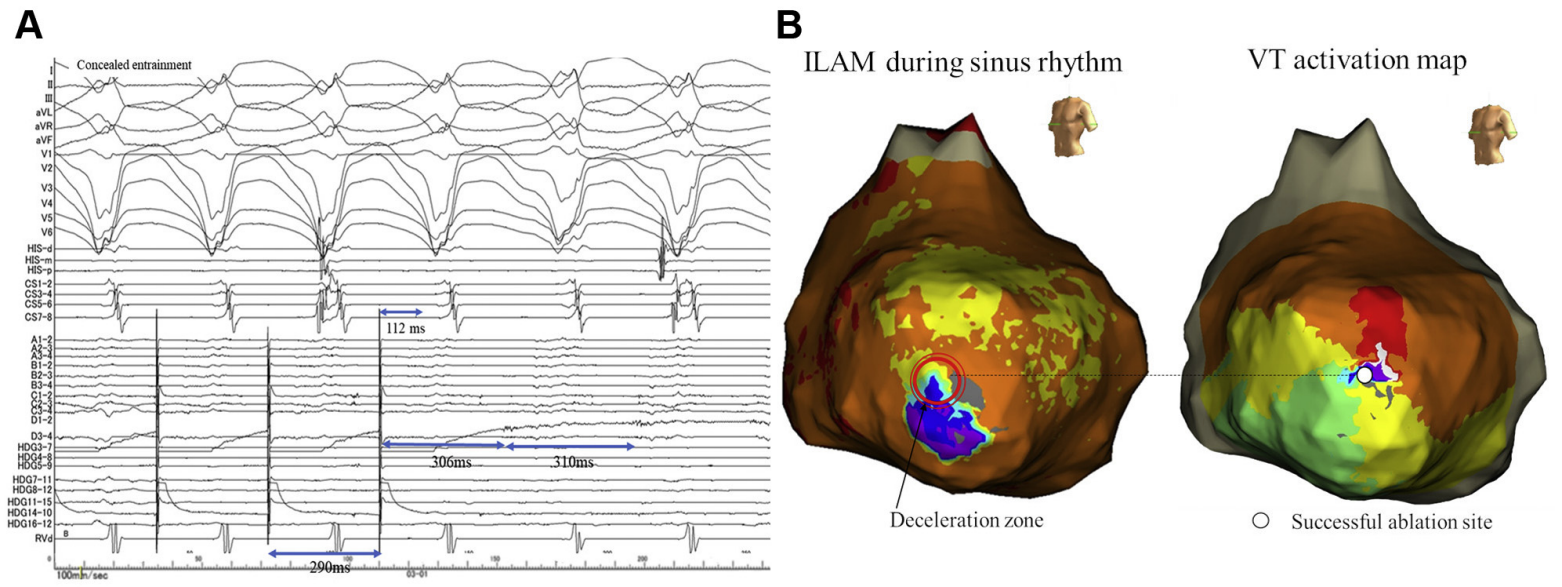
**A:** Entrainment pacing from the deceleration zone (DZ) showed concealed fusion and the difference between postpacing interval and tachycardia cycle length (PPI− TCL) was within 20 ms. Moreover, stimulus-QRS interval (112 ms) was the same as the electrogram-QRS interval during the ventricular tachycardia (VT), which represents 36% of the VT cycle length. These findings suggested that the pacing site was the isthmus site. **B:** Comparison of the location at the DZ and the successful ablation site. The left panel shows the epicardial left ventricular (LV) isochronal late activation map (ILAM) during sinus rhythm. The right panel shows the epicardial LV activation mapping during VT. The DZ around the LV apex was found to be the critical isthmus of the VT. Successful ablation site (*white dot*) was located within the deceleration zone (*red circle*).

Then, a functional substrate map was constructed using ILAM, which was reported by Aziz and colleagues.^5^ Twenty-four pairs of bipolar electrograms with the HD grid mapping catheter were recorded with automated late annotation (EnSite NavX, last deflection; Abbott, St. Paul, MN). The window of interest during sinus rhythm was set at 100 ms or 150 ms before the QRS onset. ILAM was displayed in 8 evenly distributed activation isochrones and, at this time, split at 200 ms/8 colors. ILAM showed the deceleration zone (DZ) around the LV apex, defined as a slow conduction region, and conduction velocity in the DZ is estimated as <0.6 m/s (Figure 2D). During the electroanatomical mapping, VT1 was induced. Although the VT was hemodynamically unstable, the anesthesiologist managed to maintain the blood pressure while administering intravenous catecholamine. Thus, activation mapping was performed during VT1 (Figure 3B). Interestingly, the DZ in ILAM was found to be the critical zone of VT1 (Figure 3B and Supplementary Video File). We could record the mid-diastolic potentials at the DZ during VT1. Entrainment pacing from the DZ showed concealed fusion and the difference between postpacing interval and VT tachycardia cycle length (PPI−TCL) was within 20 ms. Moreover, stimulus-QRS interval (112 ms) was the same as the electrogram-QRS interval during the VT, and represents 36% of the VT cycle length, which suggested that the pacing site was the isthmus site (Figure 3A). Then, we placed an 8F, 4-mm flexible irrigated-tip catheter with 1-4-1-mm interelectrode spacing (FlexAbility Ablation Catheter; Abbott, St. Paul, MN). The ablation was performed in this region to connect the anterior to the lateral block lines, and VT1 was immediately terminated (Figure 3B). After the ablation, VT induction was attempted with programmed ventricular stimulation from the RV apex at 2 base cycle length (400 ms and 600 ms), with up to 3 extrastimuli. However, not only VT1 but also VT2 and VT3 could not be further induced. After the discharge, the patient did not have any VT episodes during the 12-month follow-up period.

## Discussion

We report a case of successful epicardial RFCA for sustained VT in a patient with MVOHCM and AA. In our case, endocardial bipolar mapping during sinus rhythm did not reveal any abnormal electrogram findings, and the application of catheter ablation was not effective in VT termination. Therefore, the epicardial approach was performed, and widely spread low-voltage areas and late potentials were found on the epicardial side of the LV aneurysm. A conventional substrate map based on voltage and abnormal electrograms is expected to require extensive ablation lesions. However, the novel mapping technique ILAM identified the critical isthmus of VT. Recently, Aziz and colleagues^5^ demonstrated that isochronal crowding during the baseline rhythm is related to termination sites of re-entrant VT. Although multiple morphologies of VTs occurred in the present case, the remaining VTs (VT2 and VT3) were not inducible after VT1. Multiple morphologies of VTs in the same patients often occur because of different directions of re-entrant wavefront conduction around the same critical isthmus or different exit sites with the same re-entrant wavefront conductions. Anter and colleagues^6^ demonstrated that the conduction velocity–slowing area during sinus rhythm was associated with common critical zones in multiple morphologies of VTs. Therefore, ILAM might be useful in cases that are unmappable owing to hemodynamic instability or multiple VT morphologies.

Since the TCL recorded from the epicardial surface completely fulfilled the TCL of clinical VT in the present case, the re-entrant circuit was identified as a 2-dimensional circuitry completely restricted to the epicardial surface. Igarashi and colleagues^3^ reported that, in 15 patients with HCM and AA, 14 had successfully suppressed VT at the AA with the endocardial approach even if an epicardial or intramural origin of VT was suspected. However, epicardial ablation is required in the case of 2-dimensional circuit consisting only of the epicardium, as in the present case.

To the best of our knowledge, this report is the first to describe a case of successful epicardial ablation for VT with ILAM in a patient with HCM and AA.

## Conclusion

ILAM during sinus rhythm is a novel functional substrate mapping to identify the critical zone of VT. This case suggested that ILAM is useful for re-entrant VT in a patient with HCM and AA.

## Appendix Supplementary data

Supplementary Video A sparkle map of the ILAM during the sinus rhythm and activation map during the clinical VT.
